# Unlocking the mystery of the role of Vitamin D in iron deficiency anemia in antenatal women: a case control study in a tertiary care hospital in New Delhi

**DOI:** 10.1186/s12884-023-06047-w

**Published:** 2023-10-24

**Authors:** Tanishq Hitesh, Ritu Khatuja, Poonam Agrawal, Deepak Dhamnetiya, Ravi Prakash Jha, Prachi Renjhen

**Affiliations:** 1 Dr. Baba Saheb Ambedkar Medical College and Hospital, Delhi, 110085 India; 2 Department of Obstetrics and Gynaecology, Dr. Baba Saheb Ambedkar Medical College and Hospital, Delhi, 110085 India; 3 Department of Biochemistry, Dr. Baba Saheb Ambedkar Medical College and Hospital, Delhi, 110085 India; 4 Department of Community Medicine, Atal Bihari Vajpayee Institute of Medical Sciences and Dr. Ram Manohar Lohia Hospital, Delhi, New Delhi 110001 India; 5 Department of Community Medicine, Dr. Baba Saheb Ambedkar Medical College and Hospital, Delhi, 110085 India

**Keywords:** Pregnant women, Vitamin D deficiency, Anemia, Obstetrics, Maternal, Developing countries, Hemoglobin

## Abstract

**Background:**

Vitamin D deficiency and anemia are clinical conditions that coexist during pregnancy. A high prevalence of Vitamin D deficiency ranging from 50 to 94% is seen throughout the country. The aim of the study was to discover the association between Vitamin D status and iron deficiency anemia during pregnancy. Improving the vitamin D status of pregnant women is crucial to prevent iron deficiency anemia and can improve maternal and fetal outcomes.

**Methods:**

A case–control study including 94 primigravida women of age within the age group 18 to 30 years, divided into two groups: a Case Group of 48 patients with already diagnosed iron deficiency anemia (mild to moderate) and a Control Group of 46 antenatal women with normal hemoglobin levels. Data on sociodemographic, clinical characteristics, and the levels of 25(OH) Vitamin D was estimated in both the groups. The association of 25(OH)D levels and anemia was then determined using suitable statistical analysis.

**Results:**

Among pregnant women affected with anemia, 75% of women had serum Vitamin D concentrations < 20 ng/ml compared to 52.2% of women in the controls. Maternal serum vitamin D level was significantly lower in pregnant women affected with anemia (19.61 ± 13.12) as compared to control (29.43 ± 24.05); (*p* = 0.024). A positive correlation was found between hemoglobin and vitamin D levels in pregnant women (Pearson’s *r* = 0.200, *p* = 0.05).

**Conclusions:**

These findings provide evidence suggesting that Vitamin D deficiency or insufficiency during pregnancy may be a risk factor for anemia and correction of Vitamin D levels can improve hemoglobin levels. Educational efforts should be made to include safe vitamin D intake in antenatal care.

## Background

Vitamin D deficiency is recognized as a public health concern characterized by low levels of serum 25 hydroxy Vitamin D [25(OH)D] below 20 ng/ml. A high prevalence of Vitamin D deficiency ranging from 50 to 94% is seen throughout the country [1]. Major factors that are significantly associated with its deficiency include dietary habits and inadequate sunlight exposure [2]. Increasing pollution due to escalating urbanization, and lifestyle with poor outdoor activities are likely to worsen the condition [3]. In addition, its requirement further increases during pregnancy as it is crucial for maternal health, foetal skeletal growth, and optimal maternal and foetal outcomes.

Anemia is also a common problem during pregnancy, especially in developing countries. In India, as per the National Family Health Survey (NFHS-4) data, the prevalence of anemia during pregnancy is around 50% [4]. Maternal anemia has been linked with an increased risk of preterm delivery, low birth weight, intrauterine growth retardation, and adverse perinatal outcomes [5].

Vitamin D deficiency and anemia are clinical conditions that coexist during pregnancy. Studies suggest an association between Vitamin D deficiency and upregulation of mRNA expression of hepcidin, a molecule resulting in decreased level of ferroportin responsible for iron absorption, which indicates adequate Vitamin D serum levels can provide additional protection against iron deficiency in pregnant women [6]. Vitamin D receptors have already been demonstrated in bone marrow and its concentration is several 100- fold higher in bone marrow as compared to plasma [7]. Vitamin D also has an important role in erythropoiesis and it is also demonstrated that Vitamin D supplementation may improve anemia management [8].

However, the association between vitamin D deficiency and iron deficiency anemia among antenatal women remains underexplored. This study aims to bring out the potential relationship between vitamin D deficiency and iron deficiency anemia in pregnant women. Additionally, this study will provide data related to the magnitude of Vitamin D Deficiency in iron-deficient pregnant women. This study emphasized the need to improve vitamin D status in pregnant anemic women through Vitamin D supplementation along with iron supplements.

To the best of our knowledge, clinical evidence is still scarce in this field to comprehend their correlation.

### Aims and objectives

- **Primary Objective:** To assess the association of Vitamin D levels between iron-deficient anemic and nonanemic pregnant women.
- **Secondary Objectives:**
  - ◦ To assess the association of various socio-demographic factors among the study subjects.
  - ◦ To assess the association of selected maternal characteristics with iron deficiency anemia among the study subjects.
  - ◦ To assess the association of anthropometric and biochemical parameters with iron deficiency anemia among the study subjects.

## Methods

- **Design and Study Population:** A Hospital based Case Control Study was conducted among pregnant women between 28th August 2022 to 15th October 2022 in the Maternity Ward/Antenatal Clinic of the Department of Obstetrics and Gynaecology at the Dr. Baba Saheb Ambedkar Medical College and Hospital, Delhi.
  The sample size was calculated by taking the proportion of Vitamin D Deficiency in non-anemic and anemic pregnant women as 40% and 75% respectively, at 95% level of confidence and 80% power with the same number of cases and controls [9]. The minimum sample size calculated by the Fleiss formula was 31 subjects for cases and controls each. We enrolled 107 pregnant women who came for antenatal check-up or at the time of delivery and were willing to participate in the study. They were thoroughly screened by history and examination. Thirteen subjects were excluded from the study due to incomplete medical records or their samples were hemolyzed. The remaining 94 pregnant women were divided into two groups: a total of 48 antenatal women with already diagnosed iron deficiency anemia (mild to moderate) were included in the Case group and 46 antenatal women with normal hemoglobin levels were included in the Control group.
- **Eligibility Criteria:** The included subjects in both groups were primigravida women within the age group of 18 to 30 years, with confirmed singleton uncomplicated pregnancy at or after 28 weeks of gestation defined through their last menstrual period or through their first-trimester ultrasound results were included. The inclusion criteria also included the ability to provide the consent, no history of blood transfusion during current pregnancy. Exclusion criteria included pregnant women with any active comorbid disease (e.g.: thyroid diseases, chronic hypertension, seizure disorder, renal diseases, diabetes mellitus, chronic liver diseases, etc.) and anemia other than iron deficiency anemia. Patients already taking Vitamin D supplementation during pregnancy were also excluded. Patients with thalassemia (as per Mentzer Index) or history of thalassemia, sickle cell anemia, hemolytic anemia, peptic ulcer disease, gastritis, malabsorption syndrome, chronic blood loss, presence of chronic hematological diseases were also not recruited.
- **Information and Sample Collection:** Detailed history and examination were performed with regard to current pregnancy. A pre-validated and semi-structured data collection form was used to collect information on socio-economic, demographic details, personal details, pregnancy history which comprised past/ongoing medical and treatment history, iron, folic acid and Vitamin D supplement intake, regular milk consumption and information regarding present pregnancy. Antenatal investigations were noted which included blood investigations and Mentzer Index, where Mentzer Index was calculated to detect any pregnant women of β thalassemia trait. Those pregnant women who provided informed written consent, 5 ml peripheral venous blood was collected under strict aseptic conditions by a licensed laboratory technician and 2 ml of the blood was sent for. The serum was separated in a cold centrifuge within 30 min and was kept in frozen aliquots at -40 °C on the same day until analyzed. 25(OH) Vitamin D_2_ and D_3_ were measured with the help of commercially available kit using chemiluminescent immunoassay technology (CLIA) (interassay coefficient of variation, 12%). Rest of the blood was used to measure other standard blood parameters using impedance method measurement (Sysmex XP-100, Japan). The serum Vitamin D test done was an addition to the standard antenatal investigation performed in the third trimester with no extra needle prick. The 25(OH) Vitamin D levels were estimated in both groups. The association of 25(OH)D levels and anemia was then determined using suitable statistical analysis.
- **Criteria for Analysis:** Anemia in pregnant women was defined as a hemoglobin level of equal and more than 11 g/dl as normal, 10–10.9 g/dl as mild anemia, 7–9.9 g/dl as moderate anemia and 4–6.9 g/dl as severe anemia as per the guidelines of Indian Council of Medical Research (ICMR) [10].
  Vitamin D deficiency was defined as serum 25(OH)D levels less than or equal to 20 ng/ml and Vitamin D insufficiency is considered as more than 20 ng/ml but less than 30 ng/ml, while more than or equal to 30 ng/ml is considered as sufficient or normal [11].
- **Statistical Analysis:** Entries were performed and data cleaning was done from the case study form into our customized Excel Sheet (Microsoft Corp., USA). Differences in maternal characteristics and serum Vitamin D concentrations between both groups were calculated by χ2 test (for categorical variables), Mann-Whitney test (for Vitamin D concentrations), and Odds Ratio (for strength of association). All analyses were conducted using the trial version of the statistical package for social sciences (version 27.0; SPSS Inc., Chicago, IL) software, and *p* ≤ 0.05 is statistically significant.

## Results

We successfully analyzed serum 25(OH)D concentrations from all 94 samples (48 pregnant women affected with anemia and 46 controls not having anemia). The selected characteristics and sociodemographic details of the anemic women (Case) and non-anemic women (Controls) are summarized in Table 1. The mean age was 23.2 ± 3.3 years (median 22 years) for anemic pregnant women and 24.1 ± 3 years (median 24 years) for the controls. Almost all the cases were housewives (97.9%), but 8.7% of controls were employed. According to the Modified Kuppuswamy scale, nearly three-quarters (70.8%) of the cases fall under the upper lower class, while almost half (47.3%) are above the lower middle class [12]. While 56.5% of the controls have finished their intermediate class, 70.9% of the cases have not finished up to that point in their schooling. Supplementation with Iron and Folic Acid (IFA) was lacking in 43.8% of cases but present in 78.3% of controls. In contrast to the 73.9% of controls who regularly consume milk, regular milk consumption was found in only 45.8% of cases. Majority of women in both groups (89.6% in the case group and 93.5% in the control group) have more than 4 antenatal care visits to monitor the progress of their pregnancy. Nearly more than half of the studied pregnant women in both groups (58.3% in the case group and 56.5% in the control group) are non-vegetarian by diet. Among pregnant women affected with anemia, 75% of women had serum Vitamin D concentrations ≤ 20 ng/ml compared to 52.2% of women in the controls.

**Table 1:** Distribution of study subjects according to socio-demographic and maternal characteristics

Maternal Characteristics	Variable	Cases (*n* = 48) [Frequency (%)]	Controls (*n* = 46) [Frequency (%)]
**Age**	18–21	17(35.4%)	10(21.7%)
	22–25	20(41.7%)	20(43.5%)
	≥ 26	11(22.9%)	16(34.8%)
**Occupation**	Housewife	47(97.9%)	42(91.3%)
	Employed	1(2.1%)	4(8.7%)
**Education**	Till Primary School	20(41.7%)	10(21.7%)
	High School	14(29.2%)	10(21.7%)
	Intermediate	8(16.7%)	11(23.9%)
	Graduate	6(12.5%)	9(19.6%)
	Post Graduate	0	6(13.0%)
**Religion**	Hindu	41(85.4%)	40(87%)
	Others	7(14.6%)	6(13%)
**Socio economic status**	Lower	10(20.8%)	5(10.9%)
	Upper Lower	24(50.0%)	19(41.3%)
	Lower Middle	13(27.1%)	17(37.0%)
	Upper Middle	1(2.1%)	4(8.7%)
	Upper	0	1(2.2%)
**Iron / Folic Acid Supplementation**	Present	27(56.2%)	36(78.3%)
	Absent	21(43.8%)	10(21.7%)
**ANC Visits**	< 4 visits	5(10.4%)	3(6.5%)
	≥ 4 visits	43(89.6%)	43(93.5%)
**Dietary Habits**	Non-Vegetarian	28(58.3%)	26(56.5%)
	Vegetarian	20(41.7%)	20(43.5%)
**Milk Consumption**	Present	22(45.8%)	34(73.9%)
	Absent	26(54.2%)	12(26.1%)
**Serum Vitamin D Level**	Deficient	36(75.0%)	24(52.1%)
	Insufficient	3(6.3%)	9(19.6%)
	Sufficient	9(18.7%)	13(28.3%)

Independent Sample Mann Whitney U Test has been applied to check whether the distribution of BMI, Hemoglobin, RBC count, TLC, Platelet Count, MCV, MCH, MCHC, Hematocrit, and serum Vitamin D levels is same across cases and controls. We found that there is significantly lower levels of Hemoglobin (*p* = 0.000), RBC count (*p* = 0.000), MCV (*p* = 0.015), MCH (*p* = 0.000), MCHC (*p* = 0.000), and Hematocrit (*p* = 0.000) in case group as compared to the control group. The study did not find any statistical significant difference in the distribution of Body Mass Index (BMI) (*p* = 0.762), Total Leucocyte Count (TLC) (*p* = 0.264) and platelet count (*p* = 0.213) among case and control group. Maternal serum vitamin D level was significantly lower in pregnant women affected with anemia (19.61 ± 13.12) as compared to control (29.43 ± 24.05); (*p* = 0.024) [Table 2].

**Table 2:** Distribution of BMI, blood and serum parameters among anemic and non-anemic pregnant women

Measurements	Cases(*N* = 48)		Controls (*N* = 46)		*p*- value^a^
	**Mean ± SD**	**Median (IQR)**	**Mean ± SD**	**Median (IQR)**	
**BMI(kg/m**^**2**^**)**	23.94 ± 3.88	23.24 (4.73)	23.99 ± 3.17	23.33 (3.17)	0.762
**Haemoglobin (g/dL)**	9.35 ± 1.03	9.50 (1.03)	12.13 ± 0.83	12.05 (0.83)	0.000
**RBC count (× 10**^**6**^**/μL)**	3.62 ± 0.59	3.62 (0.59)	4.06 ± 0.50	4.05 (0.50)	0.000
**TLC (Total Leucocyte Count) (× 10**^**3**^**/μL)**	11.32 ± 2.99	10.45 (2.99)	11.69 ± 2.63	11.25 (2.63)	0.264
**Platelet Count (× 10**^**3**^**/μL)**	234.48 ± 103.72	222.50 (103.72)	202.46 ± 72.30	202.50 (72.31)	0.213
**MCV (fL)**	86.20 ± 11.78	85.30 (85.31)	90.76 ± 8.72	89.65 (8.72)	0.015
**MCH (pg)**	26.33 ± 4.08	26.25 (4.08)	30.22 ± 3.50	29.90 (3.50)	0.000
**MCHC (g/dL)**	30.43 ± 2.95	31.05 (2.95)	33.27 ± 1.63	33.10 (1.63)	0.000
**Hematocrit (%)**	30.64 ± 3.66	31.25 (3.66)	36.50 ± 2.49	36.45 (2.49)	0.000
**Serum Vitamin D Levels (ng/ml)**	19.61 ± 13.12	15.18 (13.13)	29.43 ± 24.05	19.59 (24.05)	0.024

*BMI* Body Mass Index, *RBC* Red Blood Cell, *MCV* Mean Corpuscular Volume, *MCH* Mean Corpuscular Hemoglobin

^a^*p*-value < 0.05 (Mann–Whitney test)

Table 3 summarizes the association of maternal characteristics and lifestyle habits among study subjects which includes iron and folic acid supplementation, Dietary habits, and milk consumption. The study did not find any statistically significant association between religion as well as dietary habits among study subjects. Subjects who are not taking Iron/Folic Acid supplementation are at 2.8 times more risk for being anemic (Odds ratio: 2.8; 95% C.I. 1.13–6.91). Subjects who are not consuming milk are at 3.35 times more risk for being anemic (Odds ratio: 3.35; 95% C.I. 1.40–7.98).

**Table 3:** Association of maternal characteristics and lifestyle habits among anemic and non-anemic pregnant women

Maternal Characteristics	Variable	Cases (*n* = 48) [Frequency (%)]	Controls (*n* = 46) [Frequency (%)]	Odds Ratio (95% CI)
**Religion**	Hindu	41(85.4%)	40(87.0%)	1
	Others	7(14.6%)	6(13.0%)	1.138 (0.352–3.683)
**Iron / Folic Acid Supplementation**	Present	27(56.3%)	36(78.3%)	1
	Absent	21(43.7%)	10(21.7%)	2.800 (1.135–6.910)
**Dietary Habits**	Non-Vegetarian	28(58.3%)	26(56.5%)	1
	Vegetarian	20(41.7%)	20(43.5%)	0.929 (0.410–2.104)
**Milk Consumption**	Present	22(45.8%)	34(73.9%)	1
	Absent	26(54.2%)	12(26.1%)	3.348 (1.404–7.986)
**Serum Vitamin D Level**	Deficient	36(75.0%)	24(52.2%)	1
	Insufficient	3(6.3%)	9(19.6%)	0.222 (0.055–0.906)
	Sufficient	9(18.7%)	13(28.2%)	0.462 (0.171–1.248)

**Fig. 1:**
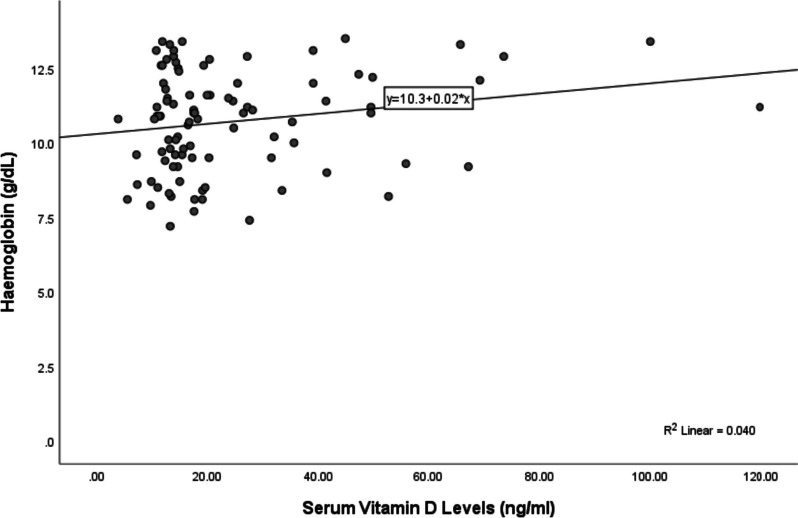
Graph showing the correlation between hemoglobin and serum vitamin D levels for pregnant women

A weak positive correlation was found between hemoglobin and vitamin D levels among study subjects (Pearson’s *r* = 0.200, *p* = 0.05) (Fig. 1).

## Discussion

Vitamin D deficiency has been linked to a variety of health consequences during pregnancy and is a global public health concern. In this hospital-based case–control study conducted on pregnant women, our main aim was to find the association between Vitamin D status and iron deficiency anemia during pregnancy. We found maternal serum Vitamin D concentrations were significantly less in women affected with anemia as compared to normal pregnant women (mean: 19.61 ng/ml in the case group vs. 29.43 ng/ml in the control group). Our results are in line with recent studies suggesting a strong association of low Vitamin D levels with anemia in pregnant women. Several studies reported low maternal vitamin D levels may be a risk factor for gestational anemia [13–17].

There are various possible explanations for the link between vitamin D deficiency and maternal anemia. Calcitriol, the active form of vitamin D, may upregulate erythropoietin receptor expression on erythroid progenitor cells and may have a proliferative impact on erythroid burst generating units, which is synergistic with erythropoietin [18, 19]. Calcitriol has an essential part in the immune function control by reducing the activity of pro-inflammatory cytokines which could be critical to its involvement in anemia prevention [20]. Studies suggest an association between Vitamin D deficiency and upregulation of mRNA expression of hepcidin, a molecule resulting in decreased level of ferroportin responsible for iron absorption, which indicates adequate Vitamin D serum levels can provide additional protection against iron deficiency in pregnant women [6]. Vitamin D receptors have already been found in bone marrow, where its concentration is hundreds folds higher than in plasma [7].

One of the most important findings of our study was the unexpectedly high prevalence of deficient Vitamin D levels among both groups (75% in case and 52.2% in control group). Hypovitaminosis D among North Indian pregnant women has also been widely reported [21–24]. The rationale might be owing to insufficient sun exposure, dark skin complexion and a lack of dietary calcium consumption, as Vitamin D synthesis initiates in the skin after sun exposure [25, 26]. High amounts of atmospheric pollution in Indian metropolitan cities could also be an important factor [27].

We also accounted for several parameters as a risk factor for maternal anemia where we reported the education status and socioeconomic status of the mother can also be a risk factor for anemia. This may be attributed to the fact that educated women belonging to middle socioeconomic status tend to prioritize self-care, actively engage in antenatal care, maintain a nutritious diet, supplement with iron and calcium, and take measures to prevent potential parasitic infections. The educational component makes the subject group more aware and responsive to new concepts and contents of public health campaigns, making them more likely to adopt good healthy living and treatment-seeking behaviour. On the other hand, uneducated and economically disadvantaged mothers are expected to lack proper nutrition [28]. This explains education and socioeconomic status are linked with maternal anemia.

Our results show a positive correlation of the effect of iron and folic acid (IFA) supplementation on maternal anemia which is consistent with various other studies [29, 30]. Although India has incorporated iron and folic acid supplementation schemes for pregnant women since 1970, these programmes have been hampered by a variety of problems and supply and coverage restrictions [31]. These schemes were relaunched in 2018 as Anemia Mukt Bharat (AMB) Abhiyan to address basic difficulties such as programme finance, execution, and last mile delivery but still the prevalence of anemia was very high during pregnancy [32].

We discovered a link between regular milk consumption and anemia. In contrast to our findings, coffee and milk have been recognized as possible iron absorption inhibitors, but only when high amounts of these items are ingested in the same meal as the iron [33]. This also shows that pregnant women are better aware of the need to take calcium and iron supplements not in the same meal. Still pregnant women should be concerned about their milk consumption as milk is an excellent source of protein and minerals like calcium, and it is linked to an increased birth weight [34].

Pregnant women identified with Vitamin D deficiency were proactively contacted, and they were provided with appropriate vitamin D supplements as per our institutional protocol. Through this study, we were able to conclude that future research should focus on more unified approaches to vitamin D testing and preventative strategies that can be integrated into already existing antenatal care settings to prevent Iron Deficiency Anemia.

A limitation of our study was the relatively small sample size and we lacked information on the iron status of the pregnant women. As a result, we were unable to explore the potential relationship between vitamin D and these markers. Further research that takes into account the above potential markers and includes a larger population size with long-term impacts is necessary and would have a significant impact on public healthcare policy.

## Conclusion

The purpose of this study was to compare vitamin D levels in iron-deficient anemia and non-anemic pregnant women. These findings provide evidence suggesting that Vitamin D deficiency or insufficiency during pregnancy may be a risk factor for anemia and correction of Vitamin D levels can improve hemoglobin levels. This study emphasized the need of improving vitamin D levels in pregnant anemic women by supplementing with vitamin D and iron. Further investigations at a larger scale on national level and multicentric trials need to be conducted to evaluate the direct relation between Vitamin D deficiency and iron deficiency anemia. Currently, Vitamin D supplementation is not recommended as a part of antenatal care programs in India. In view of the high prevalence of low Vitamin D levels in pregnancy, educational efforts need to be made to include regular safe Vitamin D and other micronutrients intake as a part of public health strategy to improve the health of both mothers and their offspring.

## Data Availability

The datasets used in the present study are available with the ICMR STS-2022 registry (ICMR STS ID: 2022–02037) and with the corresponding author on reasonable request.

## Abbreviations

25(OH)D: 25-Hydroxyvitamin D
NFHS: National Family Health Survey
MCV: Mean Corpuscular Volume
MCH: Mean Corpuscular Hemoglobin
MCHC: Mean Corpuscular Hemoglobin Concentration
HIV: Human Immunodeficiency Virus
CLIA: Chemiluminescent Immunoassay Technology
SPSS: Statistical Package for Social Sciences
ANC: Antenatal Care
BMI: Body Mass Index
IQR: Interquartile Range
RBC: Red Blood Corpuscle
TLC: Total Leucocyte Count
AMB: Anemia Mukt Bharat
ICMR: Indian Council of Medical Research
STS: Short Term Studentship

## References

[1] Aparna P, Muthathal S, Nongkynrih B, Gupta SK (2018). Vitamin D deficiency in India. J Fam Med Prim Care.

[2] Holick MF (2009). Vitamin D and health: evolution, biologic functions, and recommended dietary intakes for vitamin D. Clin Rev Bone Miner Metab.

[3] Ajmani SN, Paul M, Chauhan P, Ajmani AK, Yadav N (2016). Prevalence of vitamin D deficiency in burka-clad pregnant women in a 450-bedded maternity hospital of Delhi. J Obs Gynecol India.

[4] IIPS I. India National Family Health Survey NFHS-4 2015–16. Mumbai: IIPS and ICF. 2017:301–2.

[5] Lone FW, Qureshi RN, Emmanuel F (2004). Maternal anemia and its impact on perinatal outcome in a tertiary care hospital in Pakistan. East Mediterr Health.

[6] Braithwaite VS, Crozier SR, D’Angelo S, Prentice A, Cooper C, Harvey NC, Jones KS (2019). The effect of vitamin D supplementation on hepcidin, iron status, and inflammation in pregnant women in the United Kingdom. Nutrients.

[7] Norman AW (2006). Vitamin D receptor: new assignments for an already busy receptor. Endocrinology.

[8] Lac PT, Choi K, Liu IA, Meguerditchian S, Rasgon SA, Sim JJ (2010). The effects of changing vitamin d levels on anemia in chronic kidney disease patients: a retrospective cohort review. Clin Nephrol.

[9] El-Adawy EH, Zahran FE, Shaker GA, Seleem A (2017). Association of maternal serum 25-hydroxyvitamin D concentrations with risk of gestational anemia. Cell Physiol Biochem.

[10] Good Clinical Practice Recommendations for Iron Deficiency Anemia in Pregnancy in India. J. Obstet. Gynecol. India. 2011;61:569–71.

[11] Saleh HM, Abdel Fattah NS, Hamza HT (2013). Evaluation of serum 25-hydroxyvitamin D levels in vitiligo patients with and without autoimmune diseases. Photodermatol Photoimmunol Photomed.

[12] Saleem SM (2018). Modified Kuppuswamy scale updated for year 2018. Paripex Indian J Res.

[13] Smith EM, Alvarez JA, Martin GS, Zughaier SM, Ziegler TR, Tangpricha V (2015). Vitamin D deficiency is associated with anemia among African Americans in a US cohort. Br J Nutr.

[14] Thomas CE, Guillet R, Queenan RA, Cooper EM, Kent TR, Pressman EK, Vermeylen FM, Roberson MS, O’Brien KO (2015). Vitamin D status is inversely associated with anemia and serum erythropoietin during pregnancy. Am J Clin Nutr.

[15] Kaludjerovic J, Vieth R (2010). Relationship between vitamin D during perinatal development and health. J Midwifery Womens Health.

[16] Xia LI, Yao XI, Ge SO, Zhi-peng YU, Ling-ling WE, Ju-ying JI, Wei-min LI (2019). Correlation between vitamin D concentration and gestational anemia. Xi'an jiao tong da xue xue bao. Yi xue ban.

[17] Marei E, Elmaghraby D, Gad AA (2017). Vitamin D assessment in iron deficiency anemic pregnant women and their newborns. Egypt J Radiation Sci Appl.

[18] Aucella F, Scalzulli RP, Gatta G, Vigilante M, Carella AM, Stallone C (2003). Calcitriol increases burst-forming unit-erythroid proliferation in chronic renal failure. Nephron Clin Pract.

[19] Alon DB, Chaimovitz C, Dvilansky A, Lugassy G, Douvdevani A, Shany S, Nathan I (2002). Novel role of 1, 25 (OH) 2D3 in induction of erythroid progenitor cell proliferation. Exp Hematol.

[20] Bikle D (2009). Nonclassic actions of vitamin D. J Clin Endocrinol Metab.

[21] Sachan A, Gupta R, Das V, Agarwal A, Awasthi PK, Bhatia V (2005). High prevalence of vitamin D deficiency among pregnant women and their newborns in northern India. Am J Clin Nutr.

[22] Sahu M, Bhatia V, Aggarwal A, Rawat V, Saxena P, Pandey A, Das V (2009). Vitamin D deficiency in rural girls and pregnant women despite abundant sunshine in northern India. Clin Endocrinol.

[23] Sharma S, Kumar A, Prasad S, Sharma S (2016). Current scenario of vitamin D status during pregnancy in north Indian population. J Obstet Gynecol India.

[24] Arora S, Goel P, Chawla D, Huria A, Arya A (2018). Vitamin D status in mothers and their newborns and its association with pregnancy outcomes: experience from a tertiary care center in Northern India. J Obstet Gynecol India.

[25] Libon F, Cavalier E, Nikkels AF (2013). Skin color is relevant to vitamin D synthesis. Dermatology.

[26] Holick MF, Smith E, Pincus S (1987). Skin as the site of vitamin D synthesis and target tissue for 1, 25-dihydroxyvitamin D3: use of calcitriol (1, 25-dihydroxyvitamin D3) for treatment of psoriasis. Arch Dermatol.

[27] Agarwal KS, Mughal MZ, Upadhyay P, Berry JL, Mawer EB, Puliyel JM (2002). The impact of atmospheric pollution on vitamin D status of infants and toddlers in Delhi. India Arch Dis Childhood.

[28] Siteti MC, Namasaka SD, Ariya OP, Injete SD, Wanyonyi WA (2014). Anemia in pregnancy: prevalence and possible risk factors in Kakamega County. Kenya Sci J Pub Health.

[29] Sanghvi TG, Harvey PW, Wainwright E (2010). Maternal iron–folic acid supplementation programs: evidence of impact and implementation. Food Nutr Bull.

[30] Yakoob MY, Bhutta ZA (2011). Effect of routine iron supplementation with or without folic acid on anemia during pregnancy. BMC Pub Health.

[31] Vijayaraghavan K, Brahmam GN, Nair KM, Akbar D, Pralhad RN (1990). Evaluation of national nutritional anemia prophylaxis programme. Indian J Pediatr.

[32] Kishore S, Singh M, Jain B, Verma N, Gawande K, Kishore S, Aggarwal P, Verma SK (2020). A study to assess prevalence of anemia among beneficiaries of Anemia Mukt Bharat Campaign in Uttarakhand. J Family Med Primary Care.

[33] Pereira RC, Diniz AD, Ferreira LO (2004). New findings on iron absorption conditioning factors. Revista Brasileira de Saúde Materno Infantil.

[34] Mannion CA, Gray-Donald K, Koski KG (2006). Association of low intake of milk and vitamin D during pregnancy with decreased birth weight. CMAJ.

